# The unseen pandemic: treatment delays and loss to follow-up due to fear of COVID

**DOI:** 10.1186/s44158-021-00032-5

**Published:** 2022-01-28

**Authors:** Sharon Einav, James Tankel

**Affiliations:** 1 General Intensive Care Unit of the Shaare Zedek Medical Centre and the Hebrew University Faculty of Medicine, Jerusalem, Israel; 2 Division of Thoracic and Upper Gastrointestinal Surgery, Montreal General Hospital - McGill University Health Centre, Montreal, Quebec, Canada

**Keywords:** Viruses, Metal disorders, Infections, Role

## Abstract

**Background:**

Fear of contracting SARS-CoV-2 has transformed public interaction with healthcare professionals and hospitals alike. In turn, this has resulted in a collateral impact on patients’ health across medical and surgical paradigms. Understanding the causative factors of this fear, and tackling it head on, is vital to return to pre-pandemic levels of healthcare.

**Main body:**

In this editorial, we explore the evidence base behind the fear of healthcare professionals and facilities that has developed during the course of the SARS-CoV-2pandemic. We also reflect on the ways in which these fears have affected the general public. In so doing, we review a recent article from Montalto et al. that has explored fear of SARS-CoV-2 among patients undergoing surgery in Italy.

**Conclusion:**

While fear of SARS-CoV-2 is uncommon among surgical patients, there are still those who delay or avoiding seeking medical care due to fear of transmission. Physicians must lead the fight against this fear in a hope to regain the trust of the public.

Since first being designated a pandemic, nosocomial infection with SARS-CoV-2 has been abundantly described in the medical literature [1, 2]. With social distancing becoming the norm, avoiding transmission translated into the fear *of others* as well as the fear *for others* [3]. This newfound fear affected the perception of healthcare facilities in three ways. Firstly, as hospitals became the battleground on which the fight against SARS-CoV-2 was unfolding, non-essential services were suspended and the public urged to stay away unless necessary. Secondly, the straining of healthcare resources led to preemptive avoidance of healthcare services in an attempt to prevent an additional increase in the overall workload. Finally, hospitals became seen as a place where the risk of SARS-CoV-2 transmission was high. In this editorial, we will discuss the evidence base of the fear that has underpinned these changes and explore the impact that this has had on the health of the wider public. Finally, we will discuss a recent article that has focused on the fear of SARS-CoV-2 among patients undergoing elective surgery across hospitals in Italy and consider what, as healthcare professionals, must be done to regain the trust of the public.

The perception of healthcare facilities as “high risk” areas has not been entirely misplaced. Pre-vaccination multicenter, observational data (66,184 patients) showed that 1.4% of the patients screened had a positive SARS-CoV-2 PCR test. Among these, most were diagnosed during their hospitalization (62.1%) while a further 10.5% of these occurred at least 7 days after admission. Exposure to patients with hospital-acquired SARS-CoV-2 was associated with a substantial infection risk to other hospitalized patients. For susceptible patients, 1 day in the same ward as someone who was SARS-CoV-2 positive was associated with an additional 7.5 infections per 1000 susceptible patients per day (95% credible interval 5.5 to 9.5/1,000 susceptible patients/day) [4]. Post vaccination data also suggests that as many as 14% of inpatient infections are nosocomial in origin [5]. Clinical measures and theoretical models have been fervently pursued in an attempt to minimize these perceived risks [6], but the effectiveness of some of these measures is now being questioned.

Patients who become infected during hospitalization may also be more likely to have complications and die. Multicenter data from the first SARS-CoV2 surge showed that adult oncological patients infected during hospitalization were twice as likely to die in hospital as those admitted with community-acquired infection (RR = 2.14, 95% CI 1.76 to 2.61) [7]. More recently, patients with preoperative SARS-CoV-2 infection were shown to have increased rates of post-operative complications [8], respiratory failure, and death [9]. The paucity of data from the post-vaccination era regarding this possibility does little for the ability of the medical community to allay these fears.

Encounters with healthcare workers may also be viewed as a potential risk. In the early stages of the pandemic, both medical and paramedical staff who worked on SARS-CoV-2 wards lived in isolation from their families worldwide. While these measures are no longer considered necessary, by this time, healthcare workers have already experienced personal shunning by the public due to concerns regarding possible SARS-CoV-2 infection [10]. In addition to the *fear of the known*, the people *fear the unknown* of SARS-CoV-2 [3]. In fact, an early meta-analysis estimated that the prevalence of SARS-CoV-2 positive samples among those taken from healthcare workers was 11% (95% CI 7 to 15) when assessed using reverse transcription-polymerase chain reaction and 7% (95% CI: 4, 11) based on the presence of antibodies. Most healthcare workers with positive screens were working in hospital non-emergency wards during screening (43%, 95% CI 28 to 59), and 40% were asymptomatic at the time of diagnosis [11]. Vaccination status likely affects the prevalence of SARS-CoV-2 infection among healthcare workers. Hence, a more recent meta-analysis showed a lower overall pooled prevalence of SARS-CoV-2 among healthcare workers; 3.5% (95%CI 1.8–6.6) for studies based on molecular assays, 5.5% (95%CI 2.1–14.1) for studies based on serological assays, and 6.5% (95% CI 2.5–15.6) for point-of-care capillary blood tests [12]. Needless to say, the fear of SARS-CoV-2 transmission has also been significantly propagated by both media and social media.

These public concerns regarding possible SARS-CoV-2 transmission make the study by Montalto et al. ]13[ published in this edition of the Journal of Anesthesia, Analgesia and Critical Care very timely. In their survey of 2376 patients undergoing surgery in 27 Italian hospitals, almost half were concerned they may contract SARS-CoV-2 infection during hospitalization or while attending routine checkups in hospital. Patients undergoing surgery in hospitals with a lower surgical volume and with SARS-CoV-2 wards had more concerns regarding possible infection. Having an oncological disease was also independently associated with increased fear of infection. This first publication by the Italian Society of Anestheisa (SIAARTI - Società Italiana di Anestesia ed Analgesia) is a welcome addition to the literature. It highlights the importance of a holistic approach to the care of the patient undergoing surgery. However, no less than this, it also highlights by its very subject matter the added value of anesthesiologists as advocates of the patient.

Concerns regarding SARS-CoV-2 infection have led to cumulative collateral health-related damage. A significant increase in the number of out-of-hospital cardiac arrests has been associated with a decrease in cardiac-related treatments during the SARS-CoV-2 era [13]. Similarly, treatment delays related to SARS-CoV-2 in patients suffering from acute coronary syndromes have also been associated with an increase in other in-hospital major adverse cardiac events [14], lower residual left-ventricular function at the time of hospital discharge and an increase in predicted late cardiovascular mortality [15] .These findings have led to the publication of guidelines reinforcing the need to maintain pre-pandemic standard of care for both medical [16] and surgical [17] patients.

Montalto et al. surveyed those patients that arrived for surgery. No less interesting would be the data on those patients that never arrived. Surgical treatment delays due to late/non-referral and dropout from post-operative follow-up due to SARS-CoV-2 have thus far been relatively unstudied. In addition, while comorbidity was common in their cohort (prevalence of 54%), their patients were young relative to other surgical cohorts. Observational studies have shown that those most concerned regarding the possibility of transmission are the elderly and those suffering from psychiatric illnesses [18]. This cohort of patients was under-represented in the associated study.

Prior SARS-COV-2 infection was associated with a lower concern regarding SARS-CoV-2 re-infection during hospitalization [19]. Although reinfection with SARS-CoV-2 may occur, it seems uncommon, usually occurs with a different mutation of the virus [20–22], and is more common among persons that are immune suppressed [23]. Indeed, Current Centers for Disease Control recommendations are not to-quarantine/isolate patients who have recovered from previous SARS-CoV-2 infection if re-exposed to the virus. However, while patients that have survived SARS-CoV-2 infection may feel they have garnered a degree of immunity, the fear and anxiety of re-infection in this specific group of patients is poorly understood.

Patient anxiety is associated with the pathophysiology of the surgical stress response, potentially influencing wound repair, the immune response, inflammation, and perceptions of pain [24–28]. Montalto et al. identified two major factors associated with a worse perioperative emotional status: a-priori emotional vulnerability and surgery in hospitals with SARS-CoV-2 wards. Family support is a strong mediator for development of resilience in various disease states [29, 30]. Hence, the concerns expressed by the patients surveyed by Montalto et al. that they would not be able to see family members during hospitalization are highly relevant to the perioperative setting. It is reasonable to be concerned regarding the possibility of detachment from ones support system at the time of greatest need.

What does this study teach us? The SARS-CoV-2 pandemic has exposed many weaknesses in our healthcare systems. In this case, the main lesson is that the healthcare systems must learn to address these very relevant patient concerns. Gone are the days when trust in the treating physician sufficed to allay such fears. Our patients are partially informed and via the internet believe they have access to information previously considered within the professional domain only. The media also ensures that cutting edge data (often with a touch of exaggeration) is available to all. In order to improve medical outcomes, we must learn to listen to the needs our patients express outside of their purely medical care. Quality medical care can only be delivered with open communication and shared decision making.

Future studies should assess the relation between coping, resilience and surgical outcomes in the post- SARS-CoV-2 era. More data is required on the epidemiology and clinical implications of in-hospital transmission of SARS-CoV-2 particularly after mass vaccination. Finally, perioperative processes must be streamlined to ensure maximal patient protection during this vulnerable period in their life. Trust must be re-earned. It is imperative that we show our patients we are doing the best we can to earn their trust with regards to protecting them from SARS-CoV-2.

## Data Availability

Not applicable
